# Cost-utility analysis of dynamic intraligamentary stabilization versus early reconstruction after rupture of the anterior cruciate ligament

**DOI:** 10.1186/s13561-017-0143-9

**Published:** 2017-02-06

**Authors:** Martin Bierbaum, Oliver Schöffski, Benedikt Schliemann, Clemens Kösters

**Affiliations:** 1 Friedrich-Alexander-Universität Erlangen-Nürnberg (FAU), Nuremberg, Germany; 2 Department of Trauma, Hand and Reconstructive Surgery, University Hospital Münster, Münster, Germany

## Abstract

**Objectives:**

The aim of this study was to evaluate the cost-effectiveness of the dynamic intraligamentary stabilization (DIS) technique in comparison with reconstructive surgery (ACLR) in the treatment of isolated anterior cruciate ligament (ACL) ruptures from the perspective of the community of insured citizens in Germany.

**Methods:**

Because of the specific decision problem at hand, namely that with DIS the procedure has to take place within 21 days after the initial trauma, a decision tree was developed.

The time horizon of the model was set to 3 years. Input data was taken from official tariffs, payer data, the literature and assumptions based on expert opinion when necessary.

**Results:**

The decision tree analysis identified the DIS strategy as the superior one with 2.34 QALY versus 2.26 QALY for the ACLR branch. The higher QALY also came with higher costs of 5,398.05 € for the DIS branch versus 4,632.68 € for the ACLR branch respectively, leading to an ICER of 9,092.66 € per QALY. Results were robust after sensitivity analysis. Uncertainty was examined via probabilistic sensitivity analysis resulting in a slightly higher ICER of 9,567.13 € per QALY gained.

**Conclusion:**

The DIS technology delivers an effective treatment for the ACL rupture at a favorable incremental cost-effectiveness ratio.

**Electronic supplementary material:**

The online version of this article (doi:10.1186/s13561-017-0143-9) contains supplementary material, which is available to authorized users.

## Background

The main cause for a rupture of the anterior cruciate ligament (ACL) are non-contact injuries during football, basketball, soccer and downhill skiing [1]. The US alone spend over $1 billion on anterior cruciate ligament reconstructions (ACLR) annually [2, 3]. In Germany more than 30,000 ACLs are reconstructed every year leading to about 113.3 Million € in hospital costs. In addition, ACL tears lead to an array of indirect costs such as personal loss of income, government-funded injury leaves, absence from school or university and the loss of conditioning due to reduced activity [3]. Furthermore, rupture of the ACL is strongly linked to osteoarthrosis with many patients showing osteoarthritic changes and related functional disability as early as 10 to 15 years after initial injury [3]. Since mostly young people are affected, the prevention of long-term results becomes vitally important [4].

The current standard of care for an isolated rupture of the ACL consists of two different strategies. The first one is a wait and see strategy, also known as early rehabilitation with delayed reconstruction if needed. Here the patient tries to compensate the instability caused by the torn ACL with muscular training. This treatment is considered suitable for older and/or less active people. If the patient is not satisfied with the outcome, he can still choose reconstructive surgery later on [5, 6]. The second strategy is early reconstructive surgery (ACLR in the model). Hereby the torn ligament is reconstructed as early as six weeks after the trauma [3]. For surgical reconstruction a variety of procedures and techniques (i.e. single bundle, double bundle, allo- or autograft) exist, but recent studies show no significant differences regarding outcomes [2, 3, 6–8]. In practice younger patients are often treated surgically whereas older patients are more often treated conservatively due to their lesser demand to perform on a high activity level. Regarding different age groups and sex, current study data suggests that there is little difference in outcome between current treatment strategies [9, 10].

With the dynamic intraligamentary stabilization (DIS) a new treatment option became available. This technique makes use of the healing potential of the ligament. The surgical procedure is similar to the reconstruction, but instead of removing and replacing the original ligament, a supportive mechanism is set into the knee. With this technology an intraarticular stabilization of the knee is achieved which is accompanied by the healing of the augmented ligament. A spring system (with 8 mm deflection) compensates the anisometry of the anterior cruciate ligament. This mechanism fulfills the task of the original ligament for the time of healing. Besides the repair of the original ligament the technique has additional advantages. It potentially preserves the proprioceptive ability of the ligament, which may decrease the incidence of re-tears and the development of posttraumatic osteoarthrosis. Another advantage comes with the timing of the technique. Surgery needs to take place within 21 days after trauma. During the procedure, meniscal tears can be acutely repaired at the same time, increasing the probability of healing. This is especially important since available evidence suggests that the meniscal status is the main driver for the development of osteoarthrosis [3, 11–22].

Whereas before, wait and see was a viable strategy and reconstructions could be performed as needed, decision makers now face another strategy where time is critical. Thus the aim of our study is to analyze the cost-effectiveness of the dynamic intraligamentary stabilization technology in comparison to early reconstructive surgery as a benchmark of the current standard of care after the rupture of the anterior cruciate ligament. Our purpose is to provide decision makers with information for reimbursement decision concerning this new technology.

## Methods

### Setting and perspective

The target population of our study are patients with an isolated rupture of the anterior cruciate ligament with or without meniscal injury who are eligible for the treatment with the dynamic intraligamentary stabilization system according to the instructions of use [23]. Study setting is the German public healthcare sector with patients covered by the statutory health insurance. As far as surgical procedures are concerned the analysis is limited to the inpatient setting, because only a small number of patients are treated ambulatory. We chose the perspective of the community of insured citizens. It is the preferred perspective of the German HTA-body IQWiG when evaluating interventions. The perspective includes all direct costs, including reimbursable and out-of-pocket medical costs [24]. In contrast to the societal perspective it does not account for other social security costs and indirect costs.

### Input data

To gather the relevant information about the indication as well as the data to populate the model we performed a systematic literature search in the following databases: Medline/PubMed, Cochrane Library, NHS-EED, ScienceDirect Navigator and Scopus. The findings about the indication were then summarized into an influence diagram (see Additional file 1) which served as the basis for model development. Due to the strong heterogeneity of the study populations of the literature search it was not possible to consolidate the relevant data in the form of a meta-analysis. Instead we used the best evidence available (i.e. Cochrane review) as baseline values wherever possible. Findings from other sources were then used for the parameter ranges in the sensitivity analysis. Reimbursement rates were taken from the official tariffs. The model calculates patient copayments accordingly. Additional sources were statutory health fund data and hospital data. Health fund data was analyzed and contributed by one of the largest health funds in Germany. Hospital data was obtained from the participating hospitals in the DIS study. Parameter uncertainty in the model is addressed via one-way and probabilistic sensitivity analysis. Table 1 gives an overview of the input parameters and its sources.

**Table 1:** Input parameters

input parameter	baseline-value	SA/PSA	Source
cost inpatient surgery (DIS & ACLR)	3,605.09€	-	G-DRG catalogue
cost DIS (Ligamys)	1,284.00€	-	Mathys AG, Bettlach
cost monobloc removal (DIS)	398.85 €	2,190.83€	Eggli et al. (2016) [33], expert opinion, G-DRG catalogue
cost medical devices (ACLR)	532.00€	-	payer data
average costs of rehab per cycle	82.00€	-	payer data
cost medication (ACLR)	117.92€	-	official tariff (Lauer Taxe)
cost medication (DIS)	58.86€	-	official tariff (Lauer Taxe)
disutility for revision surgery	0.05	0–0.1	Mather et al. (2014) [6]
monobloc removal rate (DIS)	0.241	0.05	Henle et al. (2015) [11]
probability of revision surgery (DIS)	0.029	-	Henle et al. (2015) [11]
probability of revision surgery (ACLR)	0.025	0.0025–0.14	Janssen et al. (2012); Magnussen et al. (2010); Lind et al. (2012); Frobell et al. (2013) [1, 4, 15, 31]
days in hospital (DIS)	2	-	Henle et al. (2015) [11]
days in hospital (ACLR)	5	-	Geiger et al. (2013) [25]
discount rate	0.03	0–0.05%	german HTA guidelines [26]
number of prescriptions for rehab	2	-	payer data
hrQoL baseline (DIS)	0.85	beta dist. 0.85 +/- 0.09	study data on file
hrQoL baseline (ACLR)	0.80	beta dist. 0.80 +/- 0.11	Mather et al. (2014) [6]
hrQoL first 12 m (DIS)	0.79375	beta dist. 0.79375 +/- 0.1	study data on file
hrQoL first 12 m (ACLR)	0.79813	beta dist. 0.79813 +/- 0.11	Mather et al. (2014) [6]
hrQoL after revision (DIS)	lq_ACLR_norm		Assumption; equals the baseline hrQoL of ACLR because ACLR treatment is the revision therapy for DIS
hrQoL after revision (ACLR)	0.755	0.71–0.8	Lind/Menhert et al. (2012); Spindler et al. (2011); Lind/Lund et al. (2012); Wright et al.(2011) [15, 17, 27, 30]
time to revision surgery in months (ACLR)	21.6	9.6–33.6	Lind/Menhert et al. (2012) [15]
time to revision surgery in months (DIS)	11.1	3.5–24.3	Henle et al. (2015) [11]

### Costs

Since the costs for surgical treatment in the inpatient sector are covered by a case based lump sum (flat fee) we did not distinguish between different surgical approaches like single-bundle or double-bundle technique or the use of allografts vs. autografts. In the outpatient sector the situation is quite similar as well because the operational procedures are covered by a case based lump sum. Differences only occur in the coverage of the implant, which might be reimbursed in some cases [25].

All prices are reported in 2014 Euros. Cost data is based on sources assessed between 2012 and 2014, hence there is no need for adjusting unit costs. The initial treatment costs such as hospital charges, co-payments and rehabilitation charges are all incurred within a few months after the trauma. All other costs are discounted accordingly. Discount rate is set as 3% and varied between 0% and 5% for sensitivity analysis. Outcomes are discounted accordingly [26].

### Quality of life

Treatment effects after ACL-rupture are often measured with objective tests which do not necessarily reflect the patient’s subjective health related quality of life (hrQoL) [9, 15, 17, 27]. While many objective tests report significant differences between treatment strategies, studies assessing patient reported outcomes fail to support these findings. Therefore, we decided to use quality adjusted life years as the measure of benefit in our analysis.

To measure patient relevant outcomes, the SF-12 questionnaire was used to assess patient reported outcomes in a prospective open label study comparing DIS and ACLR. The study took place at the university hospital of Münster, Germany and was approved by the universities ethics committee. Informed consent to participate in the study was obtained from all participants. Questionnaires had to be filled out before surgery, at 6 weeks and at 6 and 12 months after surgery. Utilities were derived from the SF-12 data with use of the Short Form–6 dimensions (SF-6D) [28]. Due to the low enrollment rates in the ACLR group the number of returned questionnaires was insufficient for analysis (*n* = 9 at six months and *n* = 2 at twelve months). So utilities for the ACLR group had to be derived from the literature [6].

To make DIS study data and literature data comparable, hrQoL values were standardized for the first 12 months after injury. The input values and results are shown in Table 2. The values for DIS are taken from the study data. The values for ACLR where derived from Mather et al [6] where we assumed the utility value for an unstable knee to be equivalent to the quality of life before surgery which incidentally equals the pre-op utility value from our study data. The utility value for week 10 is directly taken from the literature. For the remaining months we linearly approximated the 0,81 value from the literature. Since no high quality data is available for hrQoL after revision surgery we assume the hrQoL not to be worse than an unstable knee in the ACLR group, which is a very conservative assumption in favor of the ACLR strategy.

**Table 2:** QALY calculation for the first year after surgery

DIS		ACLR	
Time	hrQoL	Time	hrQoL
Injury to surgery (3 weeks)	0.71	Injury to surgery (6 weeks)	0.71
Surgery to week 6	0.71 - > 0.75	Surgery to week 10	0.71 - > 0.82
Week 6 to 6 months	0.75 - > 0.81	Week 10 to 12 month	0.82 - > 0.81
6 months to 12 months	0.81 - > 0.85		
QALY for first year	0.79317		0.79813

### Model development

**Fig. 1:**
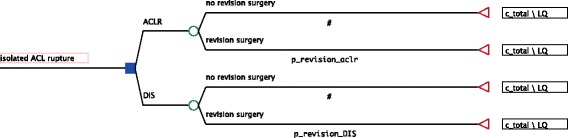
Decision Tree

In practice the decision in question has to be made within 21 days after the trauma occurs and cannot be redeemed afterwards. Given the decision problem at hand we decided to use a decision tree [29]. Figure 1 shows the structure of the decision tree.

When early reconstruction and DIS are compared, the initial physician contact and diagnostics after trauma are basically the same in both strategies, hence they are not incorporated into the model. Regarding the post-operative care, we limited the analysis to the differences between treatment strategies. Adverse events are not taken into account too. They are very rare and available study data suggests that they do not differ significantly between treatment strategies. The same states for chondral lesions, which occur seldom and do affect the outcome only in severe cases, which are not treatable with the strategies under scrutiny anyway [14, 16, 17].

Regarding revision surgery after treatment failure, the model does distinguish between treatment strategies. Revision in the DIS branch of the tree leads to costs of an ACLR and subsequently to the hrQoL of ACLR for the remaining time horizon. Revision in the ACLR branch also leads to costs of an ACLR but is assigned a lower health related quality of life for the remaining time horizon because outcomes deteriorate significantly after a second reconstruction [15, 17, 27, 30]. Re-revisions are not incorporated into the model due to high uncertainty and wide range of possible outcomes. Anyway, outcomes deteriorate even further after re-revisions irrespective of treatment strategy [15, 27].

## Results

The decision tree analysis identified the DIS strategy as the superior one with 2.34 QALY versus 2.26 QALY for the ACLR branch. The higher QALY also came with higher costs of 5,398.05€ for the DIS branch and 4,632.68€ for the ACLR branch respectively. The resulting ICER is 9,092.66€ per QALY.

**Fig. 2:**
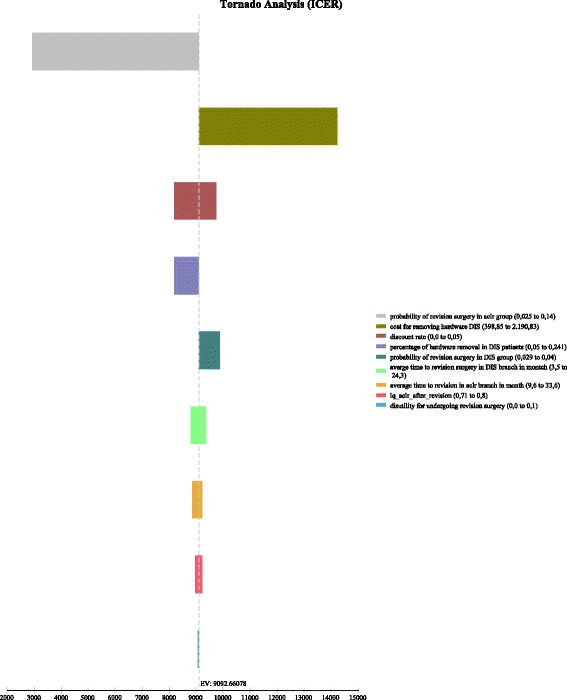
Tornado ICER

Figure 2 shows the results of the univariate sensitivity analysis. The main influencing variables are the probability of revision surgery in the ACLR group and the costs associated with the removal of the DIS monobloc. In case of the incidence of revision surgery and the timeframe within a revision surgery becomes necessary a lot of inconsistent data exists, e.g. the reported rates for revision surgery after an reconstruction of the anterior cruciate ligament lie between 2.5% and 14%. So in the base-case we used the lowest reported value of revision incidence for ACLR. So it comes with no surprise that the higher the rate of revision surgery in the ACLR group, the more favorable the DIS strategy becomes. Regarding the removal costs of the DIS monobloc it is the other way around. Basically the procedure can be performed with local anesthesia within 5 min in an ambulatory setting. Nevertheless some physicians prefer to perform the procedure in a hospital where the costs are much higher and the ICER becomes less favorable. All the remaining variables only have a marginal effect on the ICER.

**Fig. 3:**
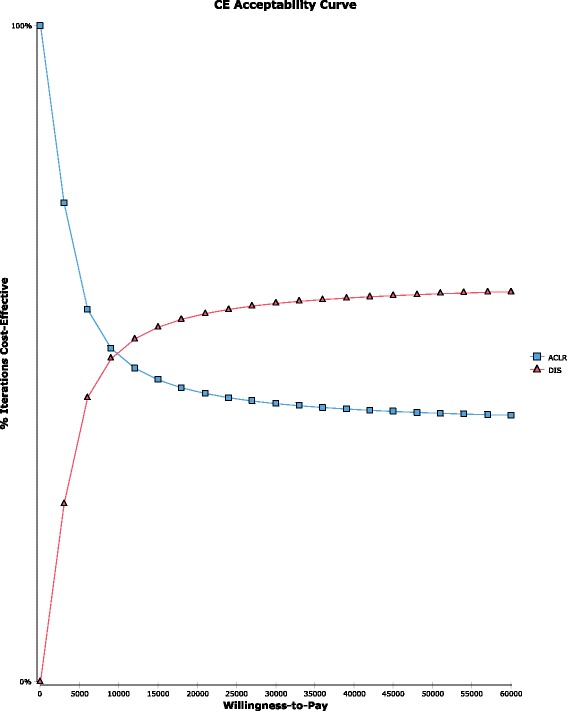
CE Acceptability Curve

Table 3 shows the results of the probabilistic sensitivity analysis for the utilities, which are in line with the decision tree results besides a slightly higher ICER of 9,567.13 €/QALY. The cost-effectiveness acceptability curve shows that beginning from a willingness to pay of 9,000 € per QALY the DIS strategy is more likely to be cost-effective (see Fig. 3).

**Table 3:** PSA analysis for effectiveness (1 Mio. runs)

	Cost (SD)	Effectiveness (SD)
ACLR	4,632.68€	2.26 QALY (+/- 0.23)
		95% CI: 1.75–2.63 QALY
DIS	5,398.05€	2.34 QALY (+/- 0.19)
		95% CI: 1.92–2.65 QALY

## Discussion

At present there is no concluding evidence available whether wait and see or early intervention is the dominant strategy [5, 31, 32]. Thus we started our modeling approach by incorporating both strategies into our model. While gathering the input data we had to realize that there is no hrQoL data available for the wait and see strategy. As a result, we limited our study to a comparison between early reconstructive surgery and dynamic intraligamentary stabilization. At first sight this seems to be a severe limitation but regarding routine practice our study still is of particular relevance. Although it cannot be known a priori if a patient will benefit from an early reconstruction, with today’s treatment strategies patients are undergoing such procedures notwithstanding. So if a patient decides against the wait and see approach the question remains which early intervention should be applied.

The scope of our analysis is limited to the inpatient setting due to data availability. Nevertheless, results should not be affected whether surgical treatment is performed out- or inpatient because overall reimbursement does not differ significantly and literature does not indicate differences in outcome. Furthermore, in Germany the majority of procedures are performed at a hospital. Moreover age and sex are not incorporated into the model. Incidence of ACL rupture has its peak between 18 and 34 years of age, so long term consequences are most likely to actually occur during the patients remaining lifetime [10, 14, 16, 17]. Studies investigating outcomes did not find clinical meaningful differences between sexes [9, 10, 17].

The time horizon of the model is limited to 3 years and cannot provide information about the long term consequences. Ultimately the patient relevant consequences are determined with the strategy chosen at the time of the ACL rupture. A knee set to develop osteoarthrosis will deteriorate further the longer the time horizon. Some of the patients will receive total knee endoprothesis, which would definitely increase costs and lower quality of life. So given today’s knowledge the results would shift in favor of the DIS strategy the longer the time horizon of the model.

Until today it is still unclear what biomechanical effects lead to osteoarthrosis after an ACL-injury, whether it is the ruptured ligament or the meniscal and cartilage injuries. If it is the latter, every form of reconstruction is meaningless regarding the long term effects of osteoarthrosis. Nevertheless, some therapies can provide better quality of life in the meantime. The uncertainty surrounding the connection between biomechanical effects and outcomes is reflected in the large span between extreme values for the probabilistic sensitivity analysis for the hrQoL. As a consequence, the probability of DIS being cost-effective for a given willingness to pay is only slightly above 50%. Therefore, payers are advised to implement health services research alongside reimbursement of the DIS technology. Irrespective of the model results, the DIS technology additionally offers some advantages in the short term. First of all, with DIS there is no need of harvesting tendons from the patient thus resulting in a lower morbidity and preserving the hamstring tendon for knee stability. Furthermore, it potentially preserves the proprioceptive ability of the ligament. Another advantage is the full weight bearing capacity after surgery allowing a more aggressive rehabilitation strategy leading to significant shorter time to work (31 vs. 65 days) respectively sports (141 vs. 185 days). Depending on the patient’s demand this might lead to a considerably higher quality of life shortly after the trauma.

## Conclusion

Overall our findings fit with the current knowledge. There is very strong evidence, that the long term outcomes are mainly dependent on the meniscal status and some studies point out that early intervention seems to have the ability to delay or prevent further degradation of the menisci [10]. Since the DIS technology by its nature offers the earliest possible moment of intervention, the probability of saving the menisci is much higher than with the other treatment strategies and consequently a lesser number of patients are likely to develop osteoarthrosis in the long term. Since until today reconstructive surgery has not been able to reduce the rate of development of osteoarthrosis, it is rather probable that a successful preventative treatment must be delivered rapidly after injury to address the early pattern of joint damage changes [3]. But not only does the DIS technology offer such an early intervention with the potential to benefit patients with an ACL rupture in long term, it also delivers a higher hrQoL in the short term at a favorable ICER of 9092.66€ when it is compared to early reconstruction.

## Additional file

Additional file 1: Influence diagram. (PDF 227 kb)
