# Supplementary figures and images for: Cost-utility analysis of dynamic intraligamentary stabilization versus early reconstruction after rupture of the anterior cruciate ligament
Source: Health Econ Rev. 2017 Feb 6;7:8. doi: 10.1186/s13561-017-0143-9 (PMC5293706; doi:10.1186/s13561-017-0143-9)

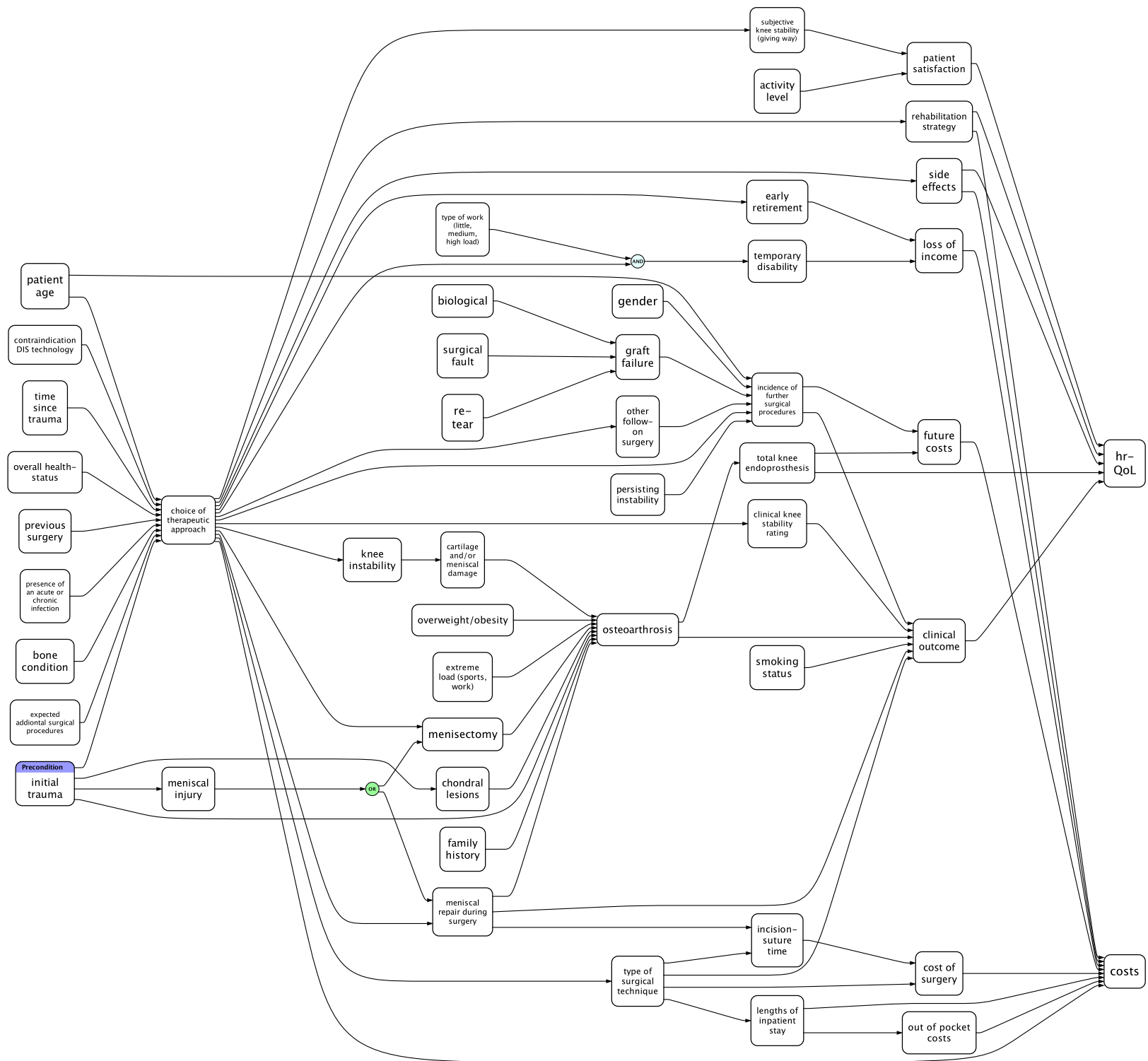

Supplement: Additional file 1: — Influence diagram. (PDF 227 kb) [file 13561_2017_143_MOESM1_ESM.pdf]
